# HDAC inhibitor valproic acid upregulates CAR in vitro and in vivo

**DOI:** 10.1186/1479-0556-5-10

**Published:** 2007-09-24

**Authors:** Blanca Segura-Pacheco, Berenice Avalos, Edgar Rangel, Dora Velazquez, Gustavo Cabrera

**Affiliations:** 1 Vectorology and Gene Therapy Laboratory, National Cancer Institute, Av. San Fernando No 22, Del. Tlalpan, CP 14080, Mexico City, Mexico

## Abstract

**Background:**

The presence of CAR in diverse tumor types is heterogeneous with implications in tumor transduction efficiency in the context of adenoviral mediated cancer gene therapy. Preliminary studies suggest that CAR transcriptional regulation is modulated through histone acetylation and not through promoter methylation. Furthermore, it has been documented that the pharmacological induction of CAR using histone deacetylase inhibitor (iHDAC) compounds is a viable strategy to enhance adenoviral mediated gene delivery to cancer cells in vitro. The incorporation of HDAC drugs into the overall scheme in adenoviral based cancer gene therapy clinical trials seems rational. However, reports using compounds with iHDAC properties utilized routinely in the clinic are pending. Valproic acid, a short chained fatty acid extensively used in the clinic for the treatment of epilepsy and bipolar disorder has been recently described as an effective HDAC inhibitor at therapeutic concentrations.

**Methods:**

We studied the effect of valproic acid on histone H3 and H4 acetylation, CAR mRNA upregulation was studied using semiquantitative PCR and adenoviral transduction on HeLa cervical cancer cells, on MCF-7 breast cancer cells, on T24 transitional cell carcinoma of the bladder cells. CAR mRNA was studied using semiquantitative PCR on tumor tissue extracted from patients diagnosed with cervical cancer treated with valproic acid.

**Results:**

CAR upregulation through HDAC inhibition was observed in the three cancer cell lines with enhancement of adenoviral transduction. CAR upregulation was also observed in tumor samples obtained from patients with cervical cancer treated with therapeutic doses of valproic acid. These results support the addition of the HDAC inhibitor valproic acid to adenoviral mediated cancer gene therapy clinical trials to enhance adenoviral mediated gene delivery to the tumor cells.

## Background

The identification of the coxsackie adenovirus receptor (CAR) and the description of its gene structure and the sequences that regulate its expression has furthered the understanding of CARs role in cellular biology, the adenoviral infection process and thus on enhancing the potential for therapeutic success in the context of adenovirus mediated cancer gene therapy [1-6]. Additionally, it has become apparent that expression of CAR is heterogeneous in diverse tumor types with implications in tumor transduction efficiency in the context of adenovirus based cancer gene therapy [7-10]. In this regard, initial findings suggest that CAR transcriptional regulation is modulated through local remodeling of the chromatin structure, mainly through histone acetylation and not through promoter methylation even though the putative promoter contains several CpG di-nucleotides [11]. Various groups have corroborated this finding utilizing various histone deacetylace inhibitors (iHDAC) to induce CAR gene expression, increase CAR presence on the surface of the tumor cells and thus enhance adenoviral transduction [12-14]. In addition to its CAR inducing potential, iHDACs posses two additional properties that would justify their addition to anti cancer gene therapy clinical trials: 1) iHDACs enhance the expression of the therapeutic gene [15-17]and 2) iHDACs display anti-neoplastic properties [18-23]. Thus, the incorporation of iHDAC compounds into the overall scheme in adenovirus mediated cancer gene therapy clinical trials seems well founded. However, reports using compounds with iHDAC properties utilized routinely in the clinic to induce the expression of CAR are pending. Valproic acid (VPA), a short chained fatty acid extensively used in the clinic to treat epilepsy and bipolar disorder has been described as an effective HDAC inhibitor [24-27]. In the present report, we studied the effect of VPA on CAR expression on HeLa cervical cancer cells, on MCF-7 breast cancer cells, on T24 transitional cell carcinoma of the bladder cells and on tumor biopsies from patients with cervical cancer treated with VPA.

## Methods

### Cell lines, cell culture and reagents

The cervical cancer cell line HeLa, the breast cancer cell line MCF-7 and the T24 transitional cell carcinoma cell line were obtained from American Type Culture Collection. Cells were grown in DMEM F12 supplemented with 10% fetal bovine serum (FBS) and 1× penicillin-streptomycin (Invitrogen, Carlsbad, CA) at 37°C and 5% CO_2_. DMEM-F12 culture media and FBS were purchased from Invitrogen (Carlsbad, CA). Trichostatin (TSA) was obtained from Santa Cruz Biotechnology (Santa Cruz, CA). Valproic acid was obtained from M.P.I Pharmaceutica GmbH, (Hamburg). OPTIMEM was obtained from Invitrogen (Carlsbad, CA)

### Recombinant Adenovirus

The adenovirus Ad-CMV-Luc encodes the luciferase gene driven by the cytomegalovirus (CMV) promoter and was a kind gift from Dr. David Curiel at the University of Alabama at Birmingham. Adenoviral preparations and titering were performed as previously described [28].

### Histone deacetylase assay

All cell lines were plated in T-150 flasks at 80% confluency. The three cell lines were treated with 5 μM TSA. HeLa cells were treated with 2 mM VPA, T24 cells 1 mM VPA and MCF7 cells 1 mM. 12 hours after treatment cells were harvested, pelleted and washed with PBS solution, RIPA buffer was added and protein quantification was performed using the bicinchoninic acid and cooper (II) sulfate method (Sigma-Aldridch St. Louis, MO). HDAC activity assay was performed using a colorimetric commercial kit from BioVision (BioVision Research Products, Mountain View, CA) following the manufacturers instructions. Briefly, 50 μg of total protein from treated cells were diluted in 85 μL of ddH_2_O; 10 μL of 10× HDAC assay buffer was added followed by the addition of 5 μL of the colorimetric substrate; samples were incubated at 37°C for 1. The reaction was stopped by adding 10 μL of lysine developer and left for an additional 30 min at 37°C. Samples were then read in an ELISA plate reader Labsystems Multiskan MS (Life Science International, Helsinki) at 405 nm. HDAC activity was expressed as percentage of activity. The kit contains negative and positive controls that consist of nuclear extract of HeLa treated or not with TSA, respectively.

### Acid extraction of proteins and western blot analysis

All cell lines were plated in T-150 flasks at 80% of confluency. The three cell lines were treated with the iHDACs as previously described. 12 hours after treatment, the cells were harvested, pelleted and washed with PBS for further acid extraction of histones with modifications [23]. Cells were then suspended in five volumes of lysis buffer [10 mM HEPES (pH 7.9), 1.5 mM MgCl_2_, 10 mM KCl, 0.5 mM DTT, and 1.5 mM phenylmethylsulfonyl fluoride] and hydrochloride acid at a final concentration of 0.2 M and subsequently lysed on ice for 30 min. After centrifugation at 11,000 × *g *for 10 min at 4°C, the cell supernatant fraction that contained acid-soluble proteins was retained. Supernatant was dialyzed against 200 mL of 0.1 M acetic acid twice for 1–2 h each and then dialyzed against 200 mL of H_2_O for 1 h, 3 h, and overnight. Dialysis was performed using a Spectra/Pore 3 Dialysis Membranes 3,500 MWCO (Spectrum Laboratories, Inc., Rancho Dominguez, CA). Five μg of acid proteins were analyzed by sodium dodecyl sulfate-polyacrylamide gel electrophoresis (SDS-PAGE)/immunoblotting with antibodies recognizing acetylated and non acetylated histones (rabbit polyclonal IgG, anti-acetyl-histone and non-acetyl-histone H4, and rabbit polyclonal IgG anti-acetyl-histone and non-acetyl-histone H3; Upstate Biotechnology, Lake Placid, NY). Protein samples were separated along with molecular weight markers (Bio-Rad, Hercules, CA) in 12% polyacrylamide gels. Gels were transferred onto 0.2 μm PVDF membranes (Bio-Rad, Hercules CA). Gel loading equivalence was confirmed by Coomassie blue stain (Sigma, St Louis, MO). Species-specific immunoglobulin G-horseradish peroxidase (IgG-HRP) secondary antibodies were purchased from Santa Cruz Biotechnology (Santa Cruz CA, USA). Blots were developed with chemiluminescent substrate (BioRad Hercules CA) and autoradiography was performed utilizing X-OMAT film (Kodak, Rochester, NY).

### CAR RT-PCR

All the cell lines were plated in T-150 flasks at 80% confluency. HeLa cells were treated with 2 mM VPA, T24 cells 1 mM VPA and MCF7 cells 1 mM. Twelve and 24 hours after treatment, the cells were harvested, pelleted and washed with PBS. RNA from drug-treated and untreated cells was obtained using TRIzol Reagent (Invitrogen, Carlsbad CA). One μg of total RNA was used for reverse transcription, which was performed with a RNA PCR Kit (Applied Biosystems, Branchburg NJ) following the manufacturer instructions. For CAR mRNA detection, the following primers were used: sense: 5'-GCCTTCAGGTGCGAGATGTTAC-3' antisense: 5'-TCGCACCCATTCGACTTAGA-3' in a total reaction volume of 20 μl. The PCR conditions were: 94°C/5 min, followed by 27 cycles at 94°C/30 s, 60°C/30 s, and 72°C/1 min. As control for the amount and integrity of the mRNA, the expression of the GAPDH gene was analyzed using the following primers sense: 5'-GAAGGTGAAGGTCGGAGTC-3' anti-sense: 5'-CAAGATGGTGATGGGATTTC-3' PCR conditions were: 94°C/5 min, followed by 27 cycles at 94°C/30 s, 55°C/30 s, and 72°C/30 s.

### Luciferase PCR

Two groups of 2 × 10^5 ^cells were plated in triplicate in 6 well plates with complete media. 24 hrs post plating, cells were treated 2 mM VPA for HeLa; 1 mM VPA for the T24 cell line and 1 mM VPA for MCF7. Twenty four hours after treatment, one group was harvested and counted. MOI was then calculated for the group that remained in culture. Cells were then transduced for 1 hour with Ad.CMV.Luc in serum free OPTIMEM (Invitrogen, Carlsbad CA, USA) with a MOI of 100 for HeLa and T24 cell lines and 10 for MCF-7 cells. After 1 hour of adenoviral transduction, the OPTIMEM was removed, cells were washed 2× with PBS, cells were then harvested and pelleted with 500 μl of lysis buffer (10 mM Tris pH 7.8, 20 mM EDTA and 0.5% SDS) for phenol-chloroform DNA extraction. The Luciferase gene was amplified using the following primers: sense 5'-ATGGAAGACGCCAAAAACATAAAG-3' antisense 5'-AAAACCGGGAGGTAGATGAGATGT-3' in a total reaction volume of 20 μl. PCR conditions were: 94°C for 5 min, followed by 25 cycles at 94°C for 30 s, 50°C for 30 s, and 72°C for 30 s and 7 min at 72°C extension. As control for the amount and integrity of the DNA, the expression of the β-actin gene was analysed using the following primers: sense 5'-ATCTGGCACCACACCTTCTACAAT-3' anti-sense 5'-CCGTCACCGGAGTCCATCA-3' PCR conditions were 94°C for 5 min, followed by 25 cycles at 94°C for 30 s, 60°C for 30 s, and 72°C for 30 s and 7 min at 72°C extension.

### Luciferase activity

Two groups of 2 × 10^5 ^cells were plated in triplicate in 6 well plates with complete media. 24 hrs post plating, cells were treated with 2 mM VPA for HeLa; 1 mM VPA for the T24 cell line and 1 mM VPA for MCF7. Twenty four hours after treatment, one group of cells was harvested and counted. MOI was then calculated for the group that remained in culture. Cells were then transduced for 1 hour with Ad.CMV.Luc in serum free OPTIMEM with the following MOIs: HeLa 100, T24 100, MCF-7 10. One hour after adenoviral transduction, OPTIMEM was removed, cells were washed 2× with PBS and complete media was then added. Forty eight hours post adenoviral transduction cells were harvested and resuspended in 50 μl of luciferase lysis buffer (Promega Inc., Madison, WI). Protein concentration was then determined using the bicinchoninic acid and cooper (II) sulfate method (Sigma-Aldridch St. Louis MO) and luciferase activity was measured as indicated by the manufacturer using a luminometer (Turner Designs, Sunnyvale, CA).

### Clinical samples and VPA dosing

**Figure 3:**
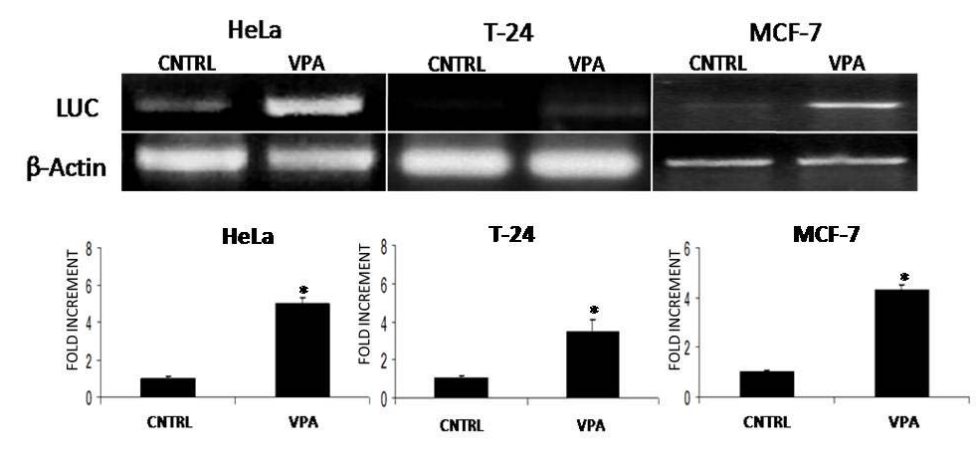
VPA mediated CAR transcriptional induction enhances adenoviral transduction and transgene expression on HeLa, T24 and MCF7 cell lines. A) Cells were treated with VPA as described in materials and methods. Twenty-four hours after treatment, cells were then transduced for 1 hour with Ad.CMV.Luc. 1 hour post adenoviral transduction, cells were washed and harvested for luciferase gene semi-quantitative PCR analysis. B) Cells were treated with VPA as described in methods. Twenty four hours after pharmacological treatment cells were then transduced for 1 hour with Ad.CMV.Luc. 48 hours post adenoviral transduction cells were harvested and assayed for luciferase activity. Asterisks indicate statistically significant changes among control vs VPA groups (p < 0.05).

RNA samples before and after VPA treatment were a kind gift from Dr. Alfonso Dueñas from a previously reported phase I clinical cervical cancer trial conducted at the National Cancer Institute, Mexico City, Mexico [23]. Briefly, biopsies were taken from areas with visible macroscopic cervical tumor using a sterile biopsy punch the day before VPA treatment. After tumor sampling, patients were started on oral valproic acid for a five-day period at 40 mg/kg. The total dose was divided in three administrations every 8 h (8 AM, 4 PM and 12 PM) per oral route in enteric-coated tablets of 200 mg. The post-treatment biopsy was taken at the sixth day post VPA treatment early in the morning, 8 to 10 hours after the last dose of VPA. Part of the biopsy was sent to the National Cancer Institutes Pathology Department for routine hematoxilin & eosin processing and observation. The remaining biopsy specimen was immediately frozen at -20°C for biological analyses. Patient 1 corresponds to patient 11, patient 2 corresponds to patient 12, patient 3 corresponds to patient 9, and patient 4 corresponds to patient 10; figure 3, reference [23].

### Statistical Analysis

Data from the luciferase reporter gene expression experiments was evaluated for statistical significance using the Students *t *test. Values less than 0.05 were considered significant.

## Results

### Valproic acid inhibits HDACs and hyperacetylates H3 and H4 histones

**Figure 1:**
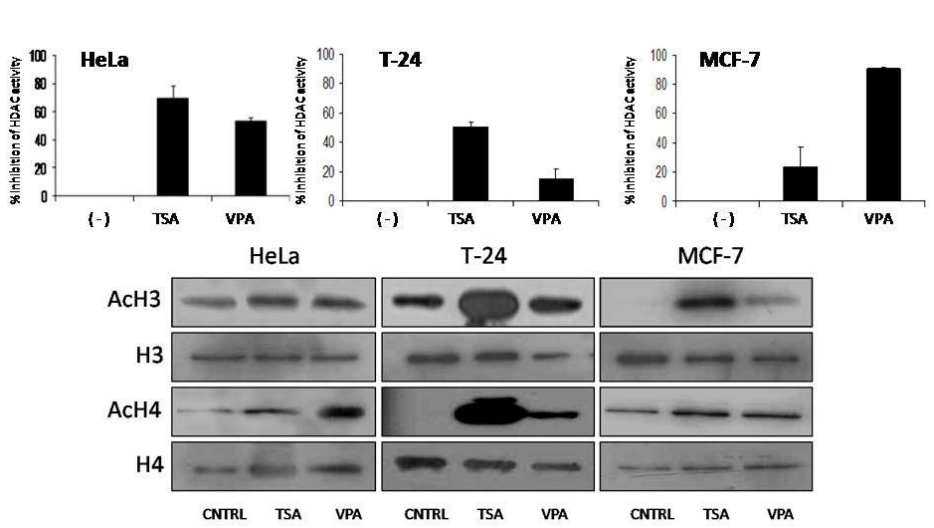
Effect of VPA on HDAC activity and histone H3 and H4 acetylation on HeLa, T24 and MCF7 cell lines. Cell lines were treated with TSA and VPA as described in "Methods". Twelve hours post pharmacological treatment cells were harvested for A) HDAC activity and B) histone H3 and H4 western blot analysis. Coomasie stained gels were used for loading control.

We initially confirmed previous reports which described VPA as an effective HDAC inhibitor. We selected a dose in which a 20% growth inhibition was observed (data not shown), we utilized a commercially available viability kit to determine the growth inhibitor concentration of VPA (MTT assay, Promega Corp, Madison, WI). Once the dose had been selected, HDAC inhibition and H3 and H4 hyperacetylation were assayed on the breast cancer cell line MCF-7, the transitional cell carcinoma of the bladder cell line T24, and cervical cancer cell line HeLa using different concentrations of VPA. Trichostatin A (TSA), a known potent HDAC inhibitor was used as a positive control. The selected doses of valproic acid for each cell line where capable of inhibiting HDAC activity within the first 12 hours as seen in figure 1a. This inhibition correlated with an increment in histone H3 and H4 acetylation. Our results suggest that valproic acid induced hypercetylation occured mainly on histone H4 while TSA induced hyperacetylation was observed on histone H3 (figure 1b).

### Valproic acid induces CAR expression *in vitro*

**Figure 2:**
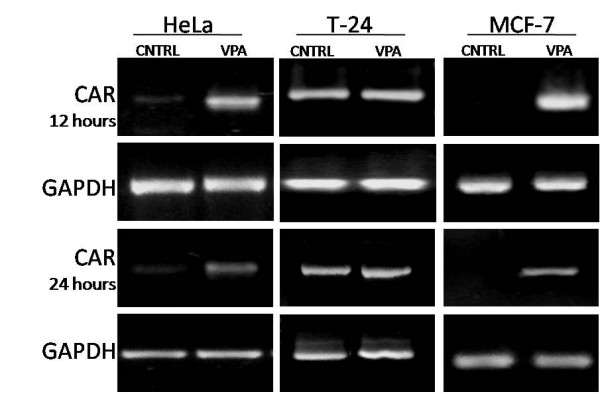
CAR mRNA transcriptional induction mediated by VPA. Given the potential use of VPA as a CAR upregulator in a clinical scenario, two potential VPA start-up times (12 or 24 hrs) prior to adenoviral gene therapy were evaluated. Twelve and twenty four hours post VPA pharmacological treatment, total mRNA was extracted, reverse transcription was performed and semi-quantitative PCR was done to assess changes on CAR mRNA levels as described in methods. The HeLa and MCF7 cancer cell lines treated with valproic acid displayed upregulation in CAR mRNA levels. The GAPDH gene was used as the loading control for semi-quantification analysis.

Given the potential use of VPA as a CAR upregulator in a clinical scenario, two potential VPA start-up times (12 or 24 hrs) prior to adenoviral gene therapy were evaluated. Twelve and twenty four hours post VPA pharmacological treatment, total mRNA was extracted, reverse transcription was performed and semi-quantitative PCR was done to assess changes on CAR mRNA levels. The HeLa and MCF7 cancer cell lines treated with valproic acid displayed a transcriptional upregulation in CAR mRNA levels as seen in figure 2. Our preliminary in vitro results suggest that patients could be started on VPA CAR induction treatment as early as 12 or 24 hours prior to adenoviral gene therapy.

### CAR upregulation enhances adenoviral transduction *in vitro*

Once determined that CAR transcription was induced by HDAC inhibition, we studied if adenoviral infection was enhanced in CAR induced cells. To this end, two sets of experiments were designed. One set of experiments determined if adenoviral genome entry was enhanced in pharmacologically induced CAR cells. The other group of experiments assessed the overall effect on reporter gene expression levels in cells in which CAR had been pharmacologically induced. The results in the first set of experiments indicate that adenoviral reporter gene entered the cells more efficiently in valproic acid treated cells when compared to the untreated control cells as seen in figure 3 panel A. These results support the results in the second set of experiments in which the levels of reporter activity correlate with the higher quantity of adenoviral genome that enter the cells in the treated groups as observed in figure 3 panel B (also see additional file 1).

### CAR mRNA increment on tumor samples

**Figure 4:**
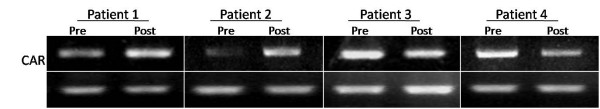
Effect of VPA on CAR transcriptional induction on tumors from patients with grade II cervical cancer treated with VPA. RNA samples before and after VPA treatment were obtained from a phase I cervical cancer trial. Pre-treatment biopsies were obtained the day before VPA treatment started. Patients were then started on oral magnesium valproate for a five-day period at 40 mg/kg. The post-treatment biopsy was taken at the sixth day post VPA treatment. RNA was extracted from the biopsy specimens for CAR RT-PCR semi-quantitative analysis. The GAPDH gene was used as loading control for semi-quantification analysis.

Since tumor transfection efficiency is a rate limiting step in adenoviral based cancer gene therapy, the clinical application of HDAC inhibitors to induce CAR expression prior to adenoviral gene delivery in order increase tumor transfection would seem rational. We thus studied VPA mediated CAR upregulation on tumor samples obtained from patients with cervical cancer before and after VPA treatment. To this end, four samples of mRNA were made available to us for CAR mRNA studies from a phase I clinical study [23]. Patients diagnosed with cervical cancer where treated with oral valproic acid as described in methods. Assessment of CAR mRNA levels was done using semi-quantitative RT-PCR as previously described. Patient 1 corresponds to patient 11, patient 2 corresponds to patient 12, patient 3 corresponds to patient 9, and patient 4 corresponds to patient 10 of figure 3, reference [23]. Results obtained from patients 1 and 2 showed an increase in CAR as seen in figure 4. The samples from patients 3 and 4 correspond to the patients with no observable changes in HDAC activity and histone acetylation levels reported previously [23] this would provide a potential explanation for the lack of CAR upregulation. The in vitro results shown in figure 2, suggest that patients could be started on VPA CAR induction treatment as early as 12 or 24 hours prior to adenoviral gene therapy. The results obtained from the clinical study suggest that patients could undergo VPA CAR induction treatment five days prior to adenoviral gene therapy. Further studies are required to establish the optimal scheme and doses for CAR upregulation in a clinical setting using VPA.

## Discussion

The success in the clinical translation of gene therapy strategies in the context of neoplastic disease depends on addressing various core issues: 1) the implementation of an effective anti-neoplastic strategy, 2) the efficient delivery of the strategy to the cells that constitute the primary tumor mass, 3) obtaining optimal transcriptional levels of the therapeutic gene and 4) expression of the putative therapeutic gene for an optimal period of time. The successful resolution of these four hurdles would be reflected on the primary tumor mass and on the control of metastatic disease. Thus, it has become clear that efficient gene delivery is a rate limiting step in cancer gene therapy [29]. Three general approaches have been devised to address the delivery issue. First, through the modification of the adenoviral fiber that would direct viral infection to a CAR independent pathway [30,31]. The second approach proposes controlling the adenoviral intratumoral dwelling time in order to allow the optimal interaction of the adenovirus with CAR and integrins in order to enhance cell transduction [32]. The third approach proposes the pharmacological induction of CAR expression. In this regard, initial studies of the CAR promoter suggest that CAR transcriptional regulation is modulated through remodeling of the chromatin structure, mainly through histone acetylation and not through promoter methylation [11]. This approach has been further supported by the use of compounds with HDAC inhibitory properties which release CAR expression from HDAC-dependent transcriptional repression. Various groups have thus shown that the pharmacological induction of CAR is a viable strategy in order to enhance adenoviral mediated gene delivery to cancer cells [12-14]. The incorporation of HDAC inhibitor drugs into the overall scheme in cancer gene therapy clinical trials would thus seem rational. This would imply the administration of routinely used pharmacological compounds in the clinic with HDAC inhibitory properties. In this regard, valproic acid is a short chained fatty acid extensively used in the clinic to treat epilepsy and bipolar disorder. VPA has been described as an effective HDAC inhibitor at therapeutic concentrations [23]. The present study demonstrates that clinically reachable serum concentrations of valproic acid increase CAR mRNA in two distinct time points; 12 and 24 hours post pharmacological treatment. These preliminary results suggest that patients undergoing adenoviral based cancer gene therapy could be started on VPA CAR induction treatment as early as 12 or 24 hours prior to adenoviral therapy. In addition to inducing CAR expression on tumor cell lines and improving the vector delivery profile in vitro, we also demonstrate that two out of four cervical cancer samples obtained from patients treated for 5 days with clinically reachable serum concentrations of valproic acid [23] increased CAR mRNA. Further studies to establish the optimal VPA doses, schemes and CAR induction windows are required in order better determine VPAs role in adenoviral based cancer gene therapy. This would be the first report documenting the pharmacological induction of CAR utilizing a HDAC inhibitor compound in humans. Furthermore, HDAC inhibitor drugs possess two additional properties that would complement the anti-neoplastic gene therapy strategy. First HDAC inhibitors are transcriptionally active compounds which enhance the expression of the therapeutic gene in the transduced cells [13,15-17,33]. Second, HDAC inhibitor drugs have per se anti-neoplastic properties [18,19].

## Conclusion

The incorporation of HDAC inhibitor drugs into the overall scheme in cancer gene therapy clinical trials would thus seem rational. Pre-clinical studies using VPA and other HDACi are required in order to further characterize doses, precise scheduling and to study possible anti-neoplastic potentiating effects.

## Abbreviations

CAR, Coxsackie and Adenovirus Receptor; VPA, Valproic acid; HDAC, Histone deacetilases; H3, Histone 3; Histone H4.

## Competing interests

The author(s) declare that they have no competing interests.

## Authors' contributions

All authors read and approved the final version of the manuscript.

AB Participated with the experimental design, carried out the HDAC activity, western blot and RT-PCR and PCR assays, data analysis and manuscript preparation.

SB Participated with experimental designs, monitored the HDAC activity, H3 and H4 western blot and RT-PCR and PCR assays and manuscript preparation and revisions.

RE Participated with the adenoviral preparations, adenovirus titering, luciferase assays and manuscript revisions.

VD Participated with the adenovirus expansion and tittering, the luciferase assays and manuscript revisions.

CG Conceptualized the project and participated with the experimental designs, data analysis and writing the manuscript.

## Supplementary Material

Additional file 1 "VPA mediated CAR transcriptional induction enhances adenoviral transgene expression on HeLa, T24 and MCF7 cell lines." Data corresponds to Figure 3, Panel B. In triplicate, cells were treated with VPA as described in methods. Twenty four hours after pharmacological treatment cells were then transduced for 1 hour with Ad.CMV.Luc. 48 hours post adenoviral transduction cells were harvested and assayed for luciferase activity. Asterisks indicate statistically significant changes among control vs VPA groups (p < 0.05). Click here for file
